# Nectin2 influences cell apoptosis by regulating ANXA2 expression in neuroblastoma: Nectin2 influences SH-SY5Y cell migration and apoptosis

**DOI:** 10.3724/abbs.2023020

**Published:** 2023-03-14

**Authors:** Shihan Zhang, Chiyi Jiang, Yan Su, Jingang Gui, Zhixia Yue, Binglin Jian, Sidou He, Xiaoli Ma

**Affiliations:** 1 Medical Oncology Department Pediatric Oncology Center Beijing Children’s Hospital Capital Medical University National Center for Children’s Health Beijing Key Laboratory of Pediatric Hematology Oncology National Key Clinical Discipline of Pediatric Oncology Key Laboratory of Major Diseases in Children Ministry of Education Beijing 100045 China; 2 Laboratory of Tumor Immunology Beijing Pediatric Research Institute Beijing Children’s Hospital Capital Medical University National Center for Children’s Health Beijing 100045 China

**Keywords:** neuroblastoma, Nectin2, apoptosis, ANXA2

## Abstract

Neuroblastoma (NB) is a pediatric cancer of the peripheral sympathetic nervous system and represents the most frequent solid malignancy in infants. Nectin2 belongs to the immunoglobulin superfamily and has been shown to play a role in tumorigenesis. In the current study, we demonstrate that serum Nectin2 level is increased in NB patients compared with that in healthy controls and Nectin2 level is correlated with neuroblastoma international neuroblastoma staging system (INSS) classification. There is a positive correlation between Nectin2 level and shorter overall survival in NB patients. Knockdown of *Nectin2* reduces the migration of SH-SY5Y and SK-N-BE2 cells and induces their apoptosis and cell cycle arrest. RNA-seq analysis demonstrates that *Nectin2* knockdown affects the expressions of 258 genes, including 240 that are upregulated and 18 that are downregulated compared with negative controls. qRT-PCR and western blot analysis confirm that ANXA2 expression is decreased in *Nectin2*-knockdown SH-SY5Y cells, consistent with the RNA-seq results. ANXA2 overexpression rescues the percentage of apoptotic NB cells induced by *Nectin2* knockdown and compensates for the impact of *Nectin2* knockdown on cleaved caspase3 and bax expressions. In addition, western blot analysis results show that ANXA2 overexpression rescues the effect of *Nectin2* knockdown on MMP2 and MMP9 expressions. The current data highlight the importance of Nectin2 in NB progression and the potential of Nectin2 as a novel candidate target for gene therapy.

## Introduction

Neuroblastoma (NB) is the most common extracranial solid malignancy in children with clinical symptoms depending on the location of the tumor [1] and has a relatively high mortality rate [2]. Indeed, NBs account for approximately 15% of all childhood cancer-related deaths due to their aggressive nature and diagnostic difficulty, despite representing only 8% of childhood malignancies [ 3, 4] . Most patients with NB die from tumor metastasis and recurrence [5]. Tumors share some essential characteristics, including excessive proliferation, disordered apoptosis and failure to differentiate [6]. A clearer understanding of the mechanism of tumor growth, metastasis and recurrence in NB is key to progress in widening treatment options.

High levels of Nectin2 expression have been observed in various tumors, including those of breast, ovarian and lung tissue [ 7, 8] . Nectin2 may play a role in tumor growth and metastasis [9], and high levels of expression have been associated with aggressive malignancy, advanced tumor stage, fast progression and poor prognosis [7]. However, the involvement of Nectin2 in NB remains unclear.

Annexin A2 (ANXA2), a member of the annexin family of proteins, is expressed on the surface of various types of cancer cells [10]. Four forms of ANXA2 exist: membrane-bound, secretory, nuclear and cytoplasmic [11]. Free cytoplasmic ANXA2 is a 36-kDa protein composed of two principal domains: the divergent NH _2_-terminal “head” and the conserved COOH-terminal protein core. The latter harbors a Ca ^2+^ binding site and a site responsible for mediating the canonical membrane binding properties [12]. ANXA2 has various cellular functions, including cell division, growth and calcium signaling [13]. In recent years, it has been shown to promote numerous processes associated with tumor progression, such as migration, invasion and proliferation [14], and to enhance multidrug resistance in NB [15].

The aim of the present study was to investigate Nectin2 expression in NB and its impact on the proliferation, apoptosis and migration of NB cells, as well as its underlying molecular mechanisms.

## Materials and Methods

### Subject recruitment and sample preparation

Ethical approval was granted by the Research Ethics Committee of Beijing Children’s Hospital (Capital Medical University, Beijing, China). Parents of all participating NB patients provided written informed consent, in accordance with the Declaration of Helsinki. A total of 38 NB patients and 33 healthy controls were recruited from Beijing Children’s Hospital, Capital Medical University, and the patients’ clinical characteristics are summarized in Table 1. Venous blood samples were collected without anticoagulant and centrifuged at 4°C for separation of serum and storage at –80°C.

**Table:** Table 1 Clinical information of neuroblastoma patients

	Frequency (%)	Nectin2 (ng/mL)	*P* value
Sex			0.003
Female	15 (39.5)	3.57 (3.42–3.64)	
Male	23 (60.5)	3.29 (2.97–3.47)	
Risk Group			0.484
Low	8 (22.2)	3.19 (2.99–3.56)	
Intermediate	6 (16.7)	3.50 (3.26–3.64)	
High	22 (61.1)	3.50 (3.26–3.64)	
LDH (U/L)			0.007
≤1500	30 (78.9)	3.43 (3.08–3.57)	
>1500	8 (21.1)	3.65 (3.57–3.67)	
NSE (ng/mL)			0.008
≤370	20 (57.2)	3.36 (2.97–3.56)	
>370	15 (42.8)	3.59 (3.42–3.66)	
Bone marrow metastasis			0.211
Yes	17 (44.7)	3.57 (3.29–3.66)	
No	21 (55.3)	3.35 (3.14–3.57)	
Bone metastasis			0.078
Yes	16 (42.1)	3.58 (3.43–3.66)	
No	22 (57.9)	3.34 (3.08–3.57)	
Lymph node metastasis			0.257
Yes	28 (73.7)	3.45 (3.11–3.60)	
No	10 (26.3)	3.57 (3.26–3.65)	
MYCN			0.793
Amplified	28 (80.0)	3.49 (3.20–3.61)	
Non- amplified	7 (20.0)	3.34 (3.15–3.63)	
Tumor size			0.068
<5 cm	9 (23.7)	3.18 (3.01–3.54)	
≥5 cm	29 (76.3)	3.49 (3.29–3.66)	
INSS			0.039
1–3	16 (42.1)	3.31 (3.06–3.52)	
4	22 (57.9)	3.58 (3.38–3.66)	

LDH: lactate dehydrogenase; NSE: neuron-specific enolase.

### Public datasets

Patient data and gene expression datasets were obtained from the R2: Genomics Analysis and Visualization Platform website ( http://hgserver1.amc.nl/cgi-bin/r2/main.cgi), which contains data from the ‘Tumor neuroblastoma Kocak-649’ and ‘Tumor neuroblastoma Asgharzadeh-249’ cohorts. All prognosis analyses were performed with R2 software.

### Determination of serum Nectin2 concentration

Blood samples were collected from patients and controls and allowed to coagulate at room temperature, centrifuged at 2810 *g* for 10 min at room temperature and immediately frozen for storage at –80°C. Nectin-2 levels were measured using a double-antibody sandwich ELISA kit (KIT10005; Sino Biological, Beijing, China) according to the manufacturer’s instructions. In brief, 100 μL of serially diluted protein standard (including zero standard) or serum was added per well, and reagents were added within 15 min. The plate was covered and incubated for 2 h at room temperature, washed 3 times with 300 μL/well wash buffer and 100 μL detection antibody at working concentration added to each well before the plate was sealed and incubated for 1 h at room temperature. Wells were aspirated and washed, and 200 μL substrate solution was added and incubated for 20 min at room temperature in the dark before 50 μL stop solution was added. Optical densities were measured within 20 min with a microplate reader at 450 nm. Zero standard optical density was subtracted, and the mean absorbance of standards was calculated and used to construct a standard curve from which target protein concentrations were estimated.

### Cell culture

The NB cell lines SK-N-BE2, SH-SY5Y and IMR-32 were purchased from the Cell Resource Center, Institute of Basic Medicine, Chinese Academy of Medical Sciences (Beijing, China) and cultured in DMEM or EMEM supplemented with 10% fetal bovine serum (Gibco, Carlsbad, USA) at 37°C with 5% CO _2_ in a humidified incubator.

### Cell transfection

*Nectin2* knockdown was achieved by transfection with lentiviral vectors containing short hairpin RNA (shRNA) sequences against Nectin2 (Nectin2 shRNA) or control (NC shRNA). Vectors were synthesized by GeneChem (Shanghai, China): sh-Nectin2#1 sequence: 5′-GCAGTGGACAGTCTGTTCAAT-3′; sh-Nectin2#2 sequence: 5′-GCAACTACACTTGCGAGTTTG-3′; sh-NC sequence: 5′-TTCTCCGAACGTGTCACGT-3′. The sh-Nectin2#2 lentiviral vector is detailed in Supplementary Data S1. ANXA2 overexpression plasmids and empty vector plasmids were synthesized by GeneChem, and details are given in the Supplementary Data S2. SH-SY5Y cell lines were cultured in 6-well plates for 24 h, and transfection was performed using Lipofectamine 2000 (Invitrogen, Carlsbad, USA) according to the manufacturer’s protocol.

### Quantitative real‐time polymerase chain reaction (qRT-PCR)

Total RNA was extracted by adding 1 mL TRIzol (Invitrogen) to cell pellets, and the lysate was transferred to a 1.5-mL microcentrifuge tube for cooling on ice for 5 min. Then, 200 μL chloroform was added, and the samples were shaken vigorously by hand for 15 s and cooled on ice for 3 min. Tubes were centrifuged for 15 min at 12,000 *g* at 4°C, the supernatant was transferred to a clean tube, and 0.5 mL isopropanol was added by mixing and shaking and placed at room temperature for 20–30 min. Tubes were centrifuged for 10 min at 12,000 *g* at 4°C, and the supernatant was discarded. The RNA pellet was resuspended in 1 mL of 75% ethanol and vortexed before centrifugation for 5 min at 7500 *g* at 4°C. The supernatant was discarded, and the RNA pellet was air dried for 5–10 min at room temperature. cDNA was synthesized using M-MLV reverse transcriptase (Promega, Madison, USA), and target sequences were amplified using Power SYBR™ Green PCR (Thermo Fisher Scientific, Waltham, USA) and primers (BGI, Beijing, China) for qRT-PCR according to the following protocol: 50°C for 2 min, 95°C for 10 min; 95°C for 15 s, 60°C for 1 min repeated for 40 cycles; followed by 95°C for 15 s. Data are presented as the fold change in gene expression, calculated by the 2 ^–ΔΔCt^ method and normalized to the endogenous reference gene *GAPDH*. Primer sequences are listed in Supplementary Table S1.

### Western blot analysis

Western blot analysis was performed to detect protein expression levels. Proteins were extracted from cell lysates using a cell lysis buffer (Beyotime, Shanghai, China) and separated by 10% SDS-PAGE followed by electrotransfer onto nitrocellulose membranes, blocking with skimmed milk at room temperature for 1 h. Membranes were incubated overnight at 4°C with primary antibodies, including anti-Nectin2 (ab135246; Abcam, Cambridge, USA), anti-GAPDH (AF1186; Beyotime), anti-β-actin (ab8226; Abcam), anti-MMP2 (AF1420; Beyotime), anti-MMP9 (AF5234; Beyotime), anti-cleaved caspase-3 (ab32042; Abcam), anti-bax (bs-0127R; Bioss, Beijing, China), anti-bcl2 (AF6285; Beyotime), anti-N-cadherin (AF0243; Beyotime), anti-E-cadherin (AF1552; Beyotime) and anti-ANXA2 (AF5115; Beyotime). Membranes were washed several times with TBST and incubated with horseradish peroxidase-conjugated secondary antibodies (KC5G5; Aksomics, Shanghai, China) at room temperature for 1 h. Protein blots were visualized using an ECL kit (P0018FS; Beyotime), and protein levels are expressed as the ratio of band optical intensity relative to that of GAPDH. Densitometry analyses were performed using ImageJ software (version 1.52; NIH, Bethesda, USA).

### RNA-seq analysis

RNA-seq analysis was performed by Novogene Company (Beijing, China). Briefly, mRNA was purified from total RNA using poly-T oligo-attached magnetic beads and fragmented using divalent cations under elevated temperature in First Strand Synthesis Reaction Buffer (5×). First strand cDNA was synthesized using random hexamer primers and M-MuLV Reverse Transcriptase, and RNA was degraded by RNase H. Second strand cDNA synthesis was performed using DNA polymerase I and dNTP. The remaining overhangs were converted into blunt ends by exonuclease/polymerase activities, 3′ ends were adenylated, and adaptors with hairpin loop structures were ligated for hybridization. Library fragments were purified with the AMPure XP system (Beckman Coulter, Beverly, USA) to preferentially select cDNA fragments of 370–420 bp in length. PCR was performed with Phusion High-Fidelity DNA polymerase, Universal PCR primers and Index (X) Primer, PCR products were purified (AMPure XP system) and library quality was assessed by an Agilent 2100 Bioanalyzer system (Agilent, Shanghai, China).

Clustering of index-coded samples was performed on a cBot Cluster Generation System using TruSeq PE Cluster Kit v3-cBot-HS (Illumina, San Diego, USA) according to the manufacturer’s instructions; library preparations were sequenced on an Illumina NovaSeq platform, and 150 bp paired-end reads were generated.

Differential expression analysis of two conditions/groups (three biological replicates per condition) was performed using the DESeq2 R package (1.20.0), which allows determination of differential expression in digital gene expression data using a model based on negative binomial distribution. The resulting *P* values were adjusted using Benjamini and Hochberg’s approach for controlling the false discovery rate.

### Flow cytometric analysis

Briefly, NB cells transfected with Nectin2 shRNA or negative control shRNA were harvested after 96 h for cell cycle analysis and processed with a Cell Cycle Staining Kit (KeyGEN BioTECH, Nanjing, China) as follows. Cells were washed with PBS, fixed with 70% ice-cold ethanol, incubated with the Cell Cycle Staining Kit for 30 min in the dark and analysed by BD FACSCanto ^TM^ flow cytometry (BD Biosciences, San Jose, USA). Additional experiments were performed to assess rates of apoptosis in which cells in logarithmic growth were harvested and stained with an Annexin V-PE/7-AAD Apoptosis Detection Kit (KeyGEN BioTECH) as follows. Cells were washed with PBS and incubated with Annexin V/7-AAD for 15 min in the dark before flow cytometric analysis.

### Wound healing assay

NB cells were transfected with Nectin2 shRNA or negative control shRNA for 96 h and seeded into 6-well cell culture plates containing serum-free DMEM. Once adhered, the cell monolayer was scratched with a 100-μL pipet tip and washed with medium. Cells were photographed under an Axio Observer D1 microscope (ZEISS, Gottingen Germany) at 0 and 24 h and quantified using ImageJ software (version 1.52; NIH). The migration rate was calculated as follows: migration rate (%)=(original wound width–wound width at 24 h)/original wound width×100%. All experiments were performed in triplicate.

### Transwell assay

Cell migration was also assessed using a Transwell chamber with 8-μm pore membranes (Corning, USA). NB cells were transfected with Nectin2 shRNA or negative control shRNA for 96 h and resuspended in serum-free medium. Cells were seeded on top of the membrane, precoated with DMEM containing 10% FBS, and DMEM was added to the lower chambers. The chamber was incubated for 24 h, and migrated cells on the underside of the membrane were fixed with 4% formaldehyde and stained with crystal violet. Cell numbers were counted under the Axio Observer D1 microscope in three random fields per well. All experiments were performed in triplicate.

### CCK8 assay

Cell proliferation was evaluated by cell counting kit-8 (CCK-8) assay. NB cells were plated and incubated in 96-well plates with 10 μL CCK-8 solution (Beyotime) added to each well for 2 h at 37°C. Absorbances were measured at 450 nm for four consecutive days to generate cell proliferation curves.

### Statistical analysis

The results are expressed as the mean±standard deviation (SD) for normally distributed data and as the median±interquartile range (IQR; 25th–75th percentiles) for nonnormally distributed data. Analyses were performed using SPSS 16.0 software. Data distribution was assessed using the Shapiro‐Wilk test. Normally distributed data were compared using Student’s *t* test or one‐way ANOVA. Nonnormally distributed unpaired data were compared using the Mann‐Whitney U test. *P*<0.05 was considered to indicate statistical significance.

## Results

### Increased Nectin2 is associated with poor prognosis of NB

**Figure 1:**
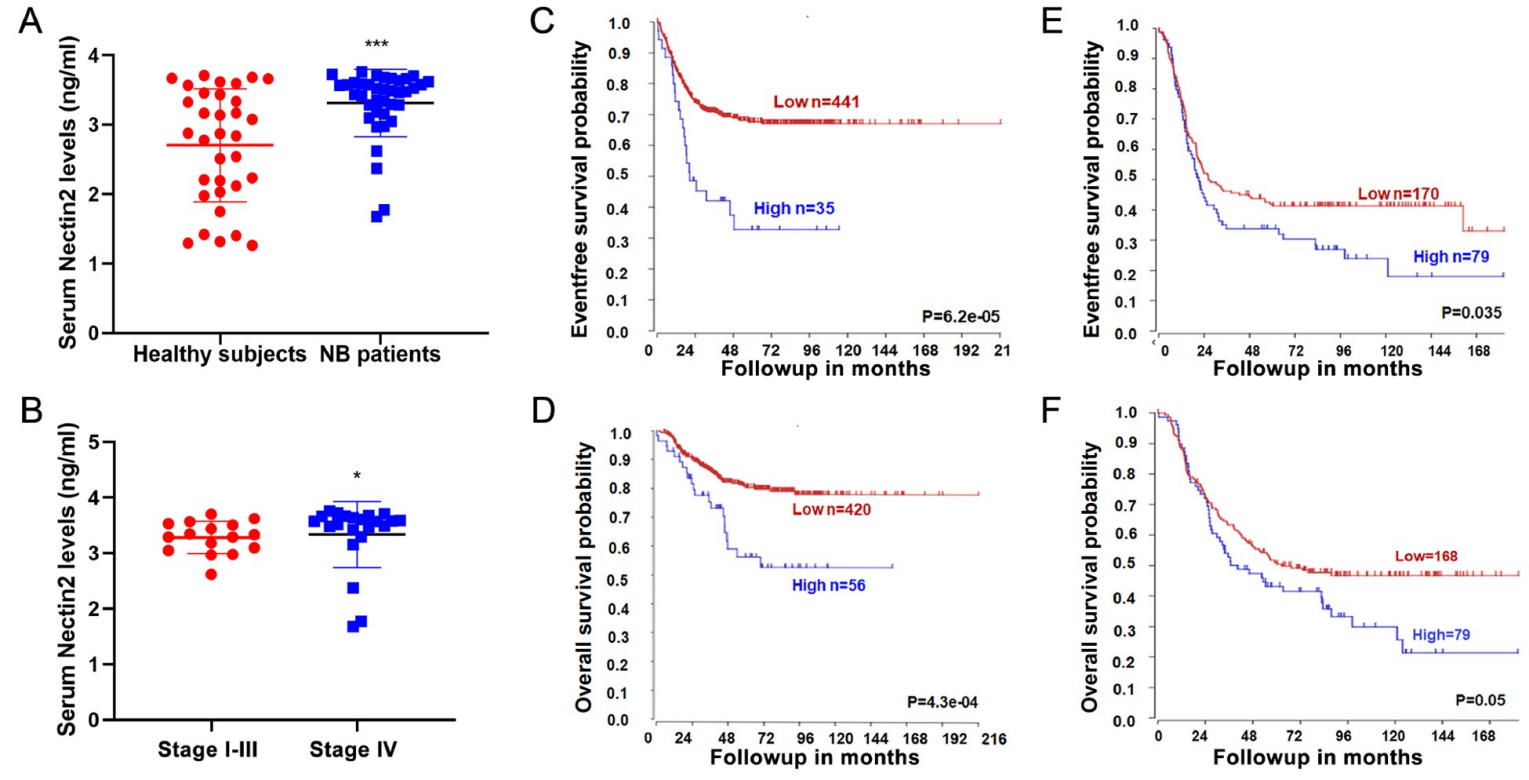
Figure 1 Elevated Nectin2 level in patients with neuroblastoma (A) Serum concentrations of Nectin2 in NB patients and healthy subjects were determined by ELISA. (B) Serum concentration of Nectin2 in stages I–III and stage IV patients was determined by ELISA. * P<0.05 vs stages I–III patients, *** P<0.001 vs healthy subjects. (C,D) Kaplan-Meier survival curve analysis according to the Nectin2 level in the Kocak-649 data. (E,F) Kaplan-Meier survival curve analysis according to the Nectin2 level in the Asgharzadeh-249 data.

ELISA measurements showed that serum level of Nectin2 was markedly increased in patients with NB compared with that in healthy controls ( Figure 1A). Nectin2 levels were correlated with tumor stage, with higher levels found in stage IV patients than in patients between stages I and III ( Figure 1B). Nectin2 expression is thus upregulated in NB patients and correlates with advanced tumor staging. Kaplan-Meier analysis of overall survival for the Kocak-649 data indicated that increased Nectin2 expression is associated with poorer prognosis ( Figure 1C,D). Similar results were observed for the Asgharzadeh-249 data ( Figure 1E,F). These results suggest that increased Nectin2 is associated with poorer outcomes in patients with NB and that the Nectin2 level may be a promising prognostic indicator.

### Knockdown of *Nectin2* inhibits the migration of NB cells

**Figure 2:**
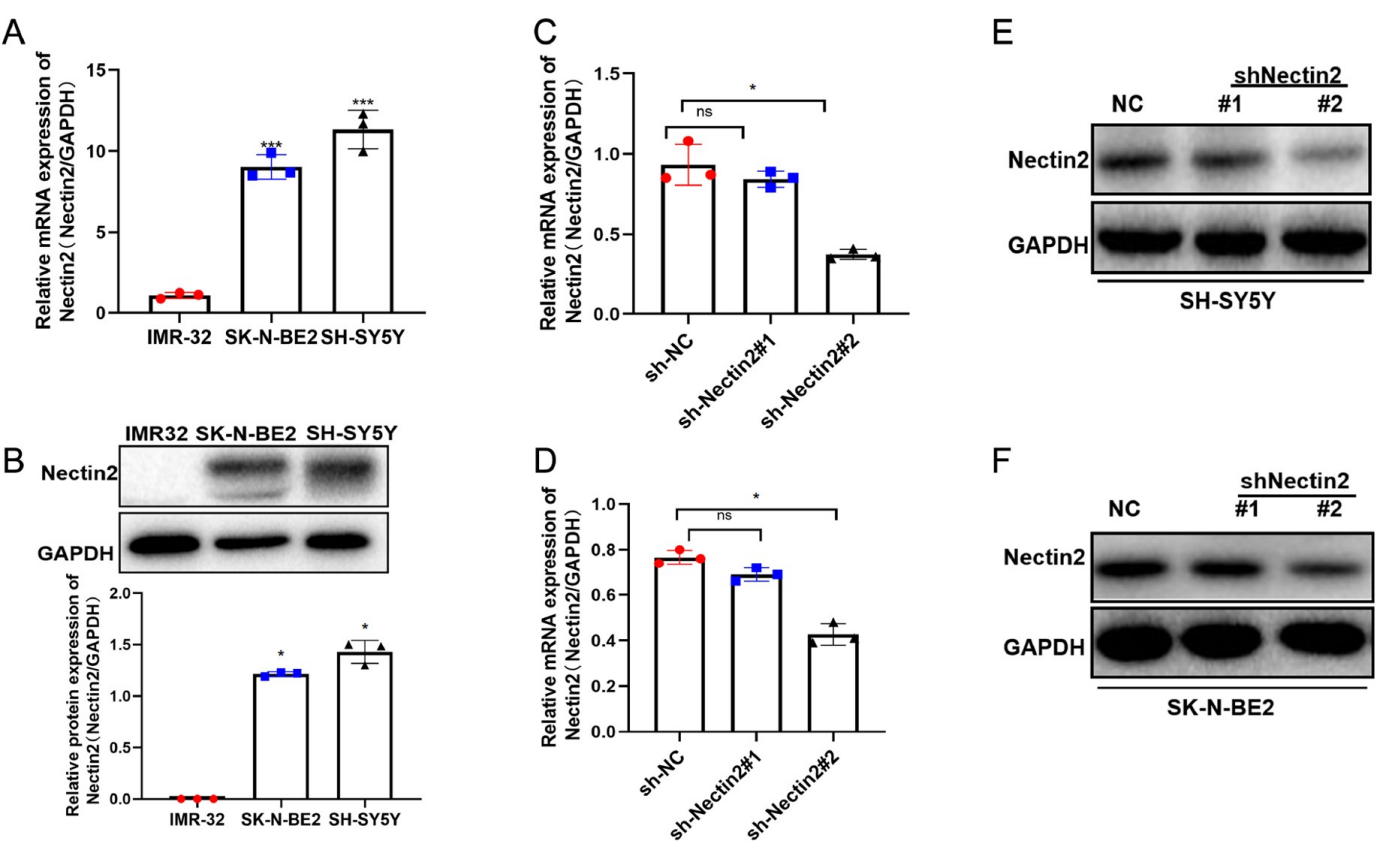
Figure 2 Nectin2 was highly expressed in neuroblastoma cells (A) Nectin2 mRNA levels measured by qRT-PCR in IMR-32, SK-N-BE2 and SH-SY5Y cells, n=3. *** P<0.001 vs IMR-32 cells. (B) Nectin2 protein levels estimated by western blot analysis in IMR-32, SK-N-BE2 and SH-SY5Y cells, n=3. * P<0.05 vs IMR-32 cells. (C,D) Nectin2 mRNA levels measured by qRT-PCR in NC shRNA- and Nectin2 shRNA-transfected SH-SY5Y and SK-N-BE2 cells, n=3. * P<0.05 vs NC shRNA group. (E,F) Nectin2 protein levels measured by western blot analysis in NC shRNA- and Nectin2 shRNA-transfected SH-SY5Y and SK-N-BE2 cells.

**Figure 3:**
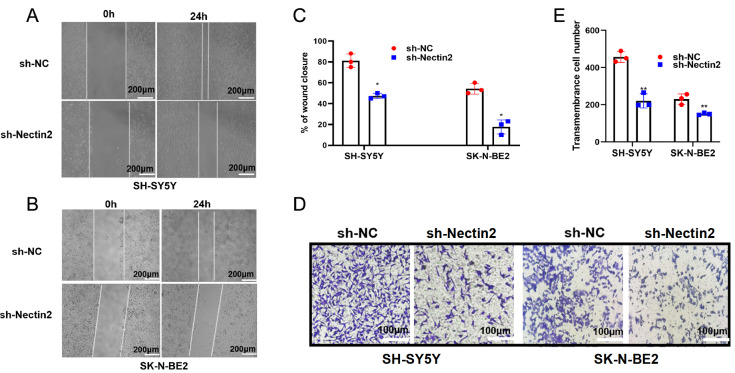
Figure 3 Knockdown of *Nectin2* inhibited the migration of SH-SY5Y and SK-N-BE2 cells (A–C) Wound healing assays of cell migration in SH-SY5Y and SK-N-BE2 cells, n=3. * P<0.05 vs NC shRNA group. (D,E) Transwell assays of cell migration in SH-SY5Y and SK-N-BE2 cells, n=3. ** P<0.01 vs NC shRNA group.

**Figure 4:**
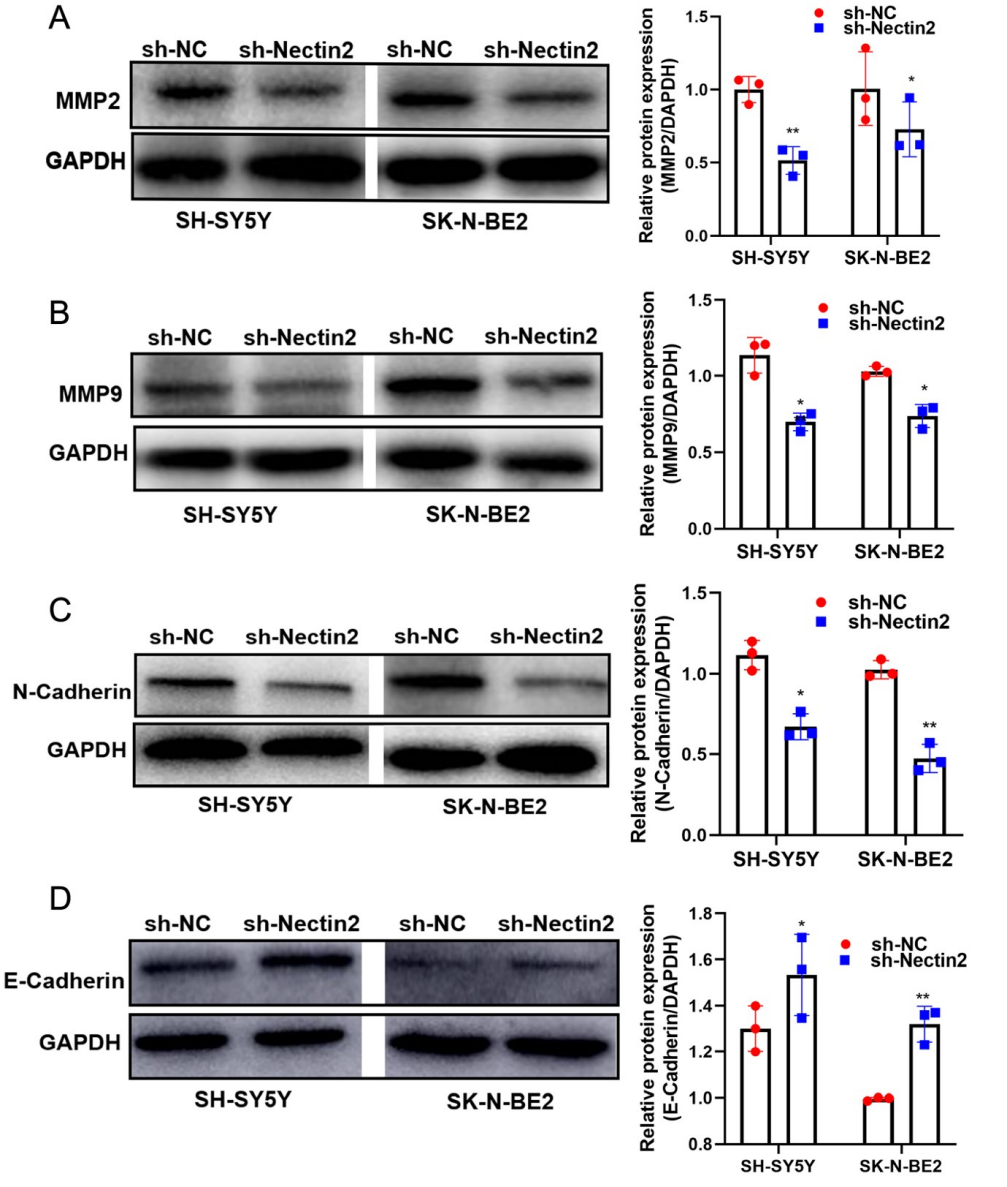
Figure 4 Knockdown of *Nectin2* inhibited the expressions of migration-related proteins (A,B) MMP2 and MMP9 protein levels were detected by western blott analysis. (C,D) N-cadherin and E-cadherin protein levels were detected by western blot anaysis, n=3. * P<0.05, ** P<0.01 vs NC shRNA group.

Analyses of Nectin2 expressions in the NB cell lines SH-SY5Y, SK-N-BE2 and IMR-32 by qRT-PCR and western blot analysis showed high expression in SH-SY5Y and SK-N-BE2 cells ( Figure 2A,B). Nectin2 shRNAs and NC shRNAs were transfected into SH-SY5Y and SK-N-BE2 cells, and the results demonstrated that shNectin2#2 had high knockdown efficiency and was selected for subsequent experiments ( Figure 2C‒F). Wound healing and transwell assays revealed that knockdown of *Nectin2* significantly suppressed the migration of NB cells ( Figure 3A‒E). The expression of the migration-related genes *MMP2*, *MMP9* and *N-cadherin* was reduced by *Nectin2* knockdown ( Figure 4A‒C), and that of *E-cadherin* was stimulated ( Figure 4D). Therefore, Nectin2 expression shows a positive correlation with NB cell migratory ability.

### *Nectin2* knockdown induces apoptosis and cell cycle arrest of NB cells

**Figure 5:**
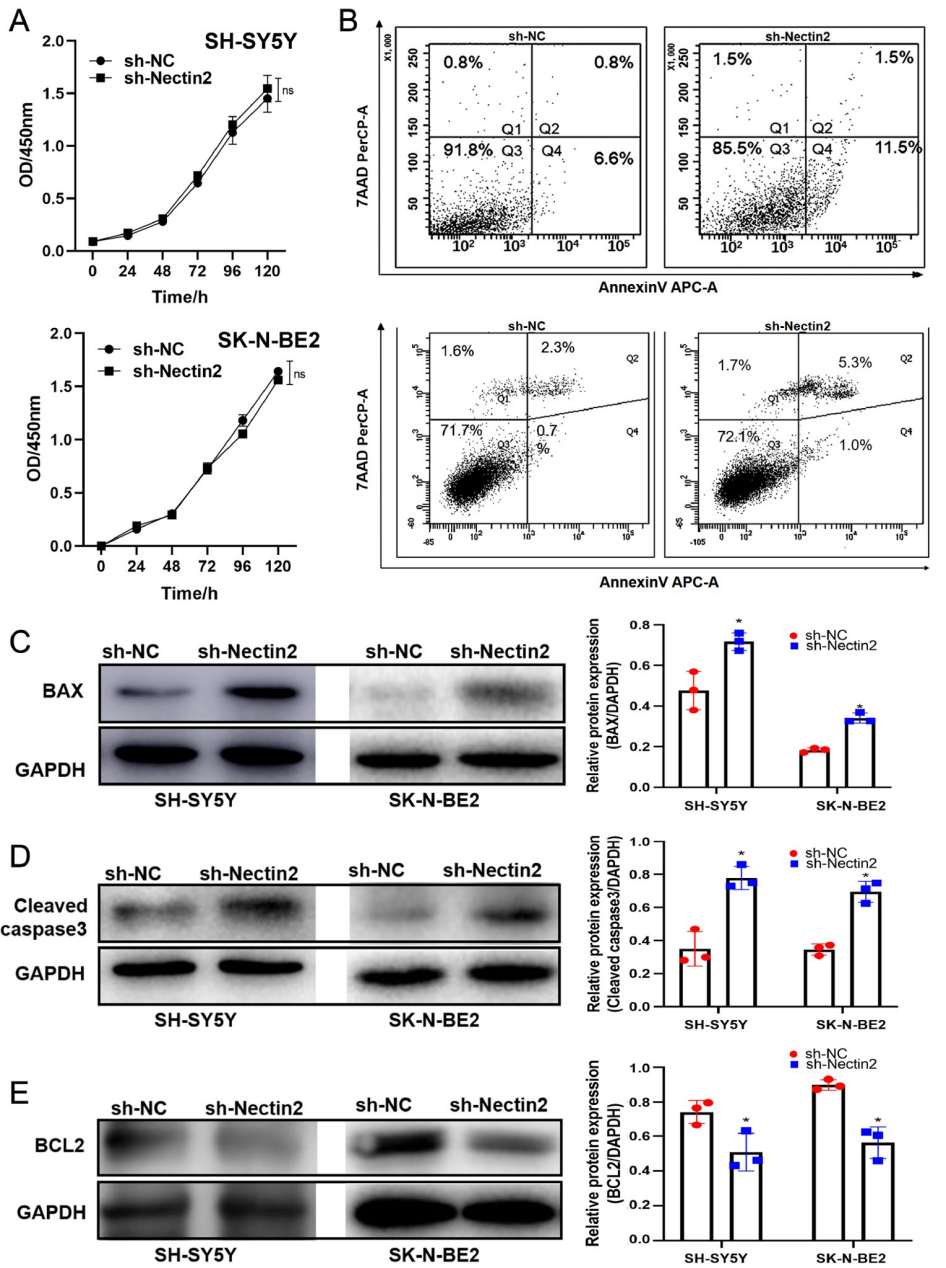
Figure 5 Knockdown of *Nectin2* induced apoptosis in SH-SY5Y and SK-N-BE2 cells (A) Cell proliferation was measured by CCK-8 assay. (B) Apoptosis was assessed by flow cytometry. (C–E) Bax, cleaved caspase3 and bcl2 expressions were measured by western blot analysis, n=3. * P<0.05 vs NC sh-RNA group.

**Figure 6:**
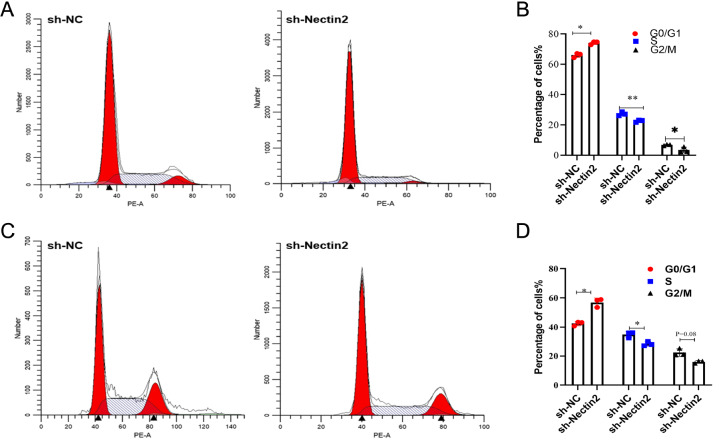
Figure 6 Knockdown of *Nectin2* arrested the cell cycle of SH-SY5Y and SK-N-BE2 cells at the G0/G1 phase (A,B) SH-SY5Y cells were stained with PI and analysed by flow cytometry, n=3. * P<0.05, ** P<0.01 vs NC sh-RNA group. (C,D) SK-N-BE2 cells were stained with PI and analysed by flow cytometry, n=3. * P<0.05 vs NC sh-RNA group.

*Nectin2* knockdown did not alter the proliferative rates of NB cells, as assessed by CCK-8 assay ( Figure 5A). However, flow cytometric analysis revealed increased percentages of apoptotic cells ( Figure 5B) and increased expressions of the apoptosis-related proteins (cleaved caspase3 and bax), while bcl2 expression was decreased ( Figure 5C‒E). Cell cycle arrest in the G0/G1 phase was detected following *Nectin2* knockdown ( Figure 6A‒D). These findings indicate that *Nectin2* knockdown induces apoptosis of NB cells and cell cycle arrest but does not affect cell proliferation.

### Nectin2 regulates ANXA2 expression in SH-SY5Y cells

**Figure 7:**
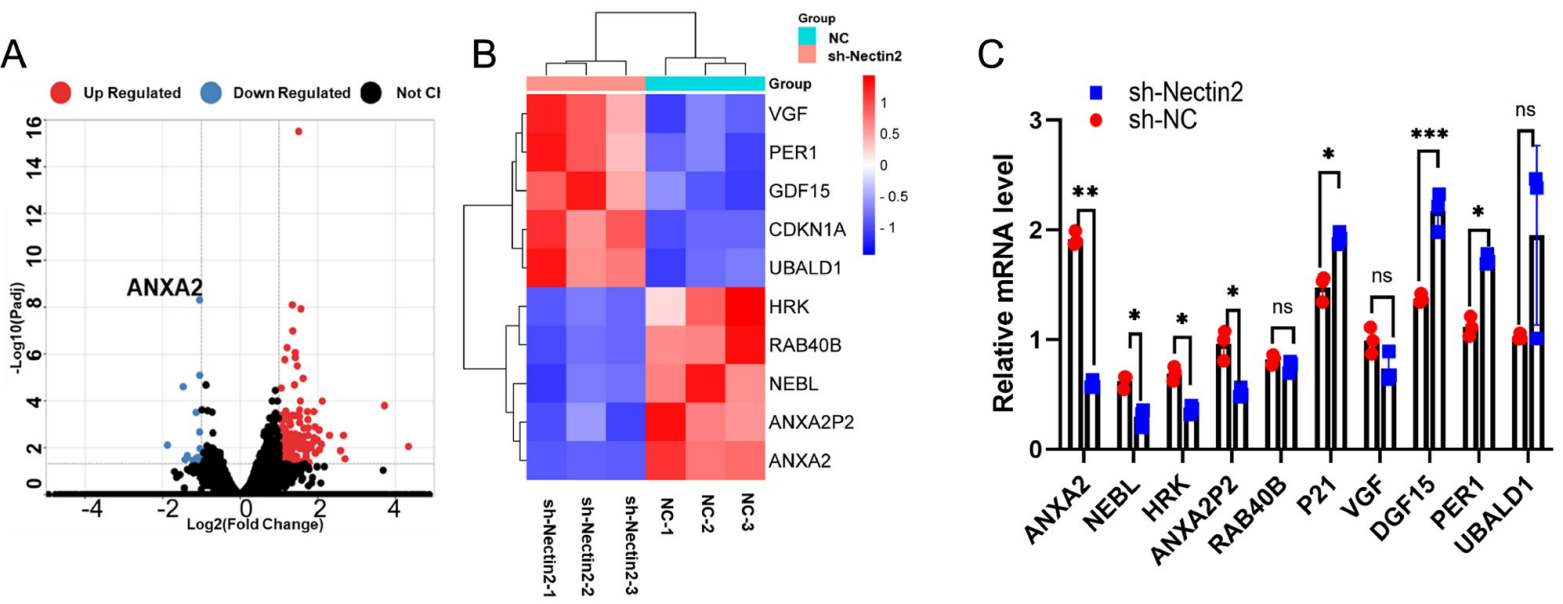
Figure 7 Altered gene expression after *Nectin2* knockdown in SH-SY5Y cells (A) Volcano map of RNA-seq of target genes after Nectin2 knockdown. (B) Heatmap image of RNA-seq of target genes after Nectin2 knockdown (Fold Change>2). Red block: upregulation; blue blocks: downregulation. (C) The top 5 differentially expressed up- and downregulated genes in the NC shRNA group and Nectin2 shRNA group were confirmed by qRT-PCR, n=3. * P<0.05, ** P<0.01, *** P<0.001 vs NC sh-RNA group.

**Figure 8:**
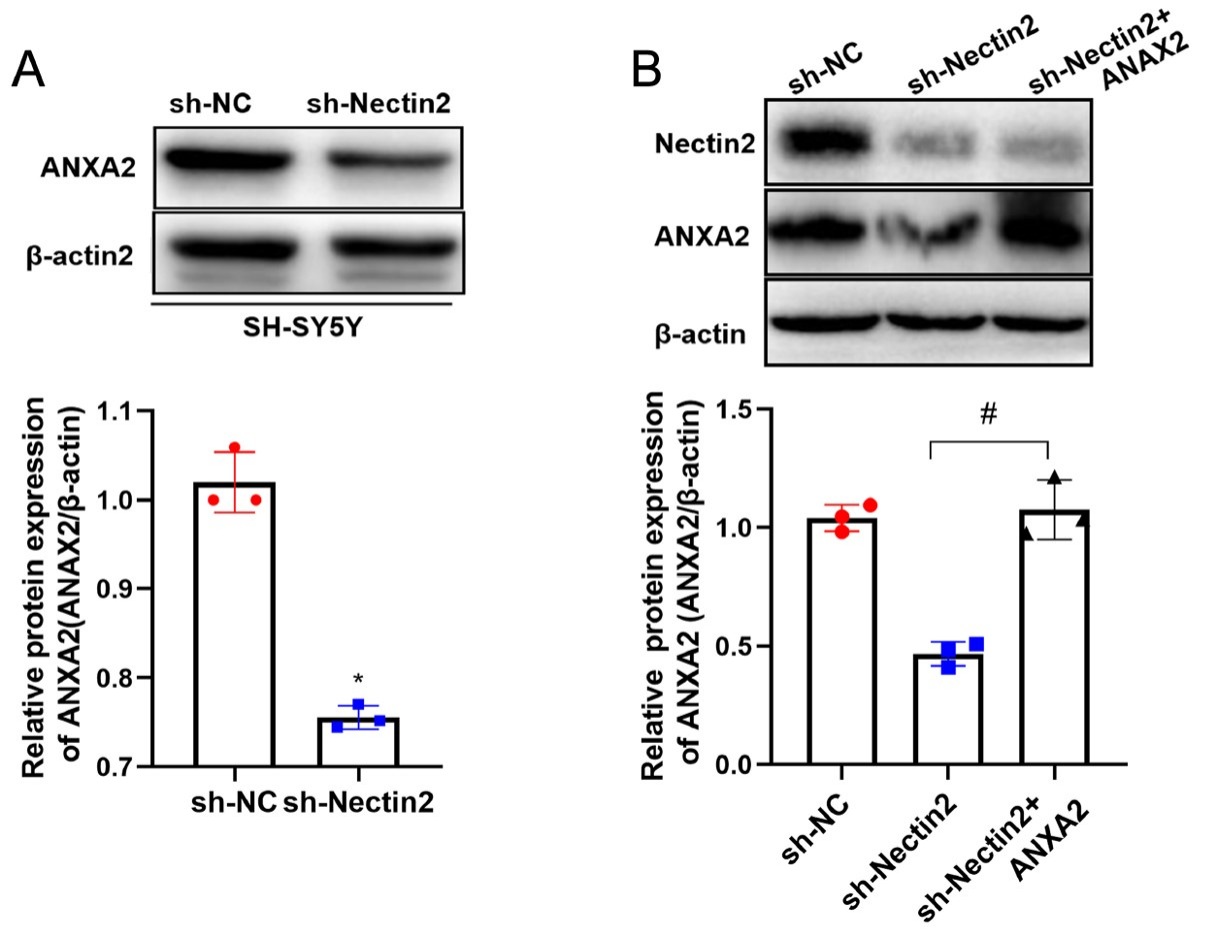
Figure 8 Knockdown of *Nectin2* decreased ANXA2 expression (A,B) ANXA2 protein levels in the NC shRNA group and Nectin2 shRNA group were detected by western blot analysis. (C,D) ANXA2 protein levels in the NC shRNA group, Nectin2 shRNA group and Nectin2 shRNA+ANXA2 group were detected by western blot analysis, n=3. * P<0.05 vs NC shRNA group; # P<0.05 vs Nectin2 shRNA group.

**Figure 9:**
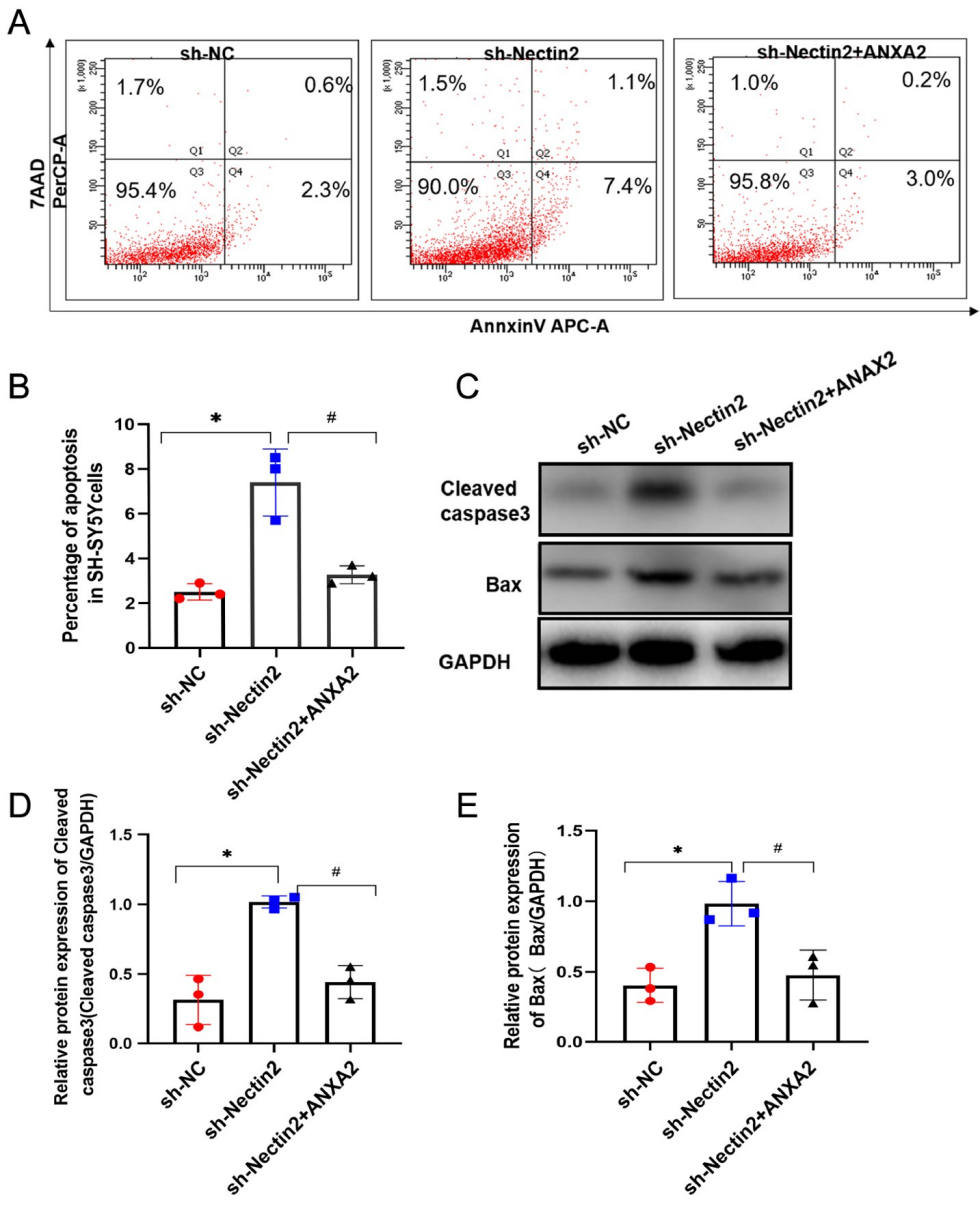
Figure 9 ANXA2 overexpression rescued the effect of *Nectin2* knockdown on apoptosis (A,B) Apoptosis was assessed by flow cytometry in the NC shRNA group, Nectin2 shRNA group and Nectin2 shRNA+ANXA2 group. (C–E) Cleaved caspase3 and bax protein levels were detected by western blot analysis, n=3. * P<0.05 vs NC shRNA group, # P<0.05 vs Nectin2 shRNA group.

**Figure 10:**
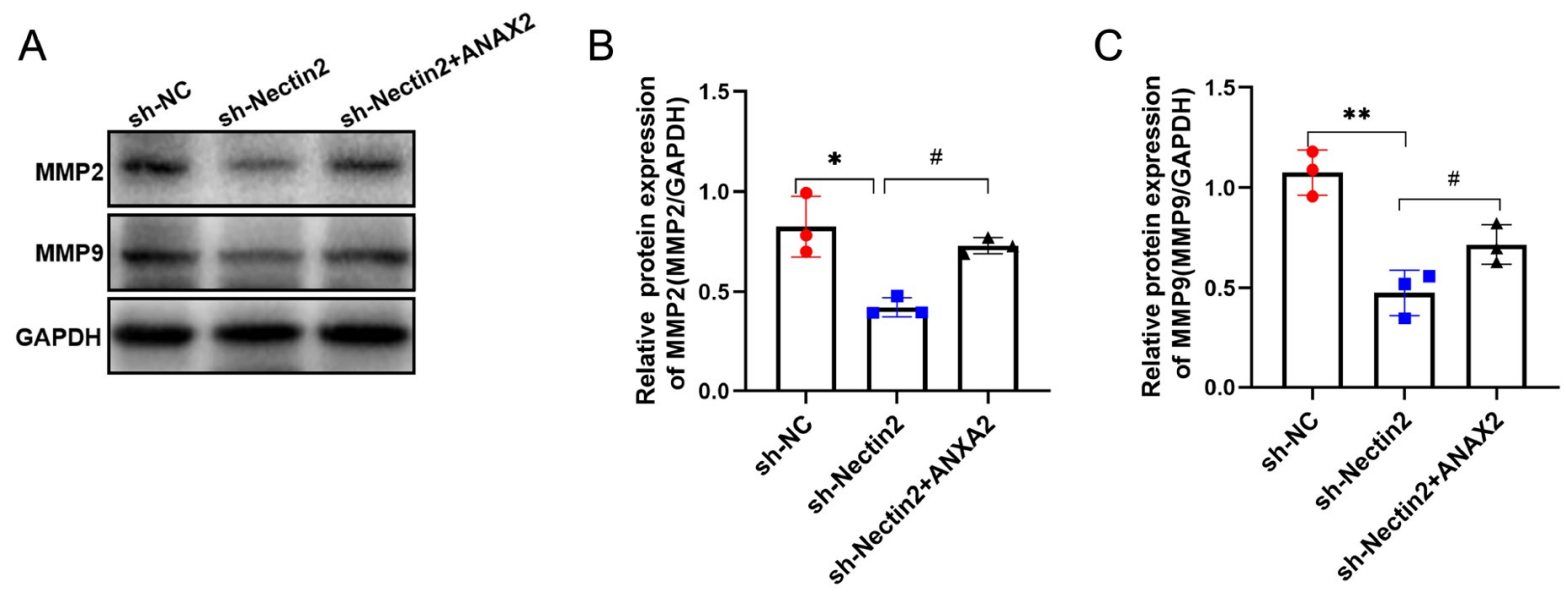
Figure 10 ANXA2 overexpression rescued the effect of *Nectin2* knockdown on cell migration (A–C) MMP2 and MMP9 levels were detected by western blot analysis, n=3. * P<0.05, ** P<0.01 vs NC shRNA group; # P<0.05 vs Nectin2 shRNA group.

*Nectin2* knockdown in SH-SY5Y cells caused 258 genes to be differentially expressed ( *P*-adj<0.05 and Fold Change>2), including 240 genes that were upregulated and 18 that were downregulated ( Figure 7A). The 5 genes showing the greatest degree of differential expression are listed in Figure 7B and Table 2, and the RNA-seq results were confirmed by qRT-PCR ( Figure 7C). *ANXA2* is a gene known to be related to NB, and its expression has been correlated with tumor metastasis and poor prognosis in NB patients [ 9, 15] . Western blot analysis confirmed that the expression of ANXA2 protein is consistent with the RNA-seq results ( Figure 8A). The ANXA2 overexpression plasmid was transfected into SH-SY5Y cells, and the results revealed that the transfection efficiency was higher than 90% ( Supplementary Figure S1). The ANXA2 overexpression plasmid was transfected into SH-SY5Y cells in which *Nectin2* had been knocked down ( Figure 8B). ANXA2 overexpression rescued the apoptotic SH-SY5Y phenotype induced by *Nectin2* knockdown ( Figure 9A,B). In addition, ANXA2 overexpression rescued the effect of *Nectin2* knockdown on cleaved caspase3 and bax expressions ( Figure 9C‒E) and on those of MMP2 and MMP9 ( Figure 10), as revealed by western blot analysis. Thus, Nectin2 is likely to influence cell apoptosis via downregulation of ANXA2 expression.

**Table:** Table 2 The top 5 differentially expressed up-regulated genes and down-regulated genes between sh-Nectin2- and sh-NC-transfected SH-SY5Y cells

Gene name	log2(Fold Change)	*P*-adj	Expression
*CDKN1A(P21)*	1.508854	3.12E–16	UP
*VGF*	1.344829	8.01E–09	UP
*GDF15*	1.568166	1.18E–08	UP
*UBALD1*	1.35533	1.03E–07	UP
*PER1*	1.208882	5.35E–07	UP
*ANXA2*	–1.04833	4.90E–09	DOWN
*NEBL*	–1.04578	8.08E–06	DOWN
*ANXA2P2*	–1.47215	2.47E–05	DOWN
*RAB40B*	–1.13624	0.000313	DOWN
*HRK*	–1.04907	0.002151	DOWN

## Discussion

The current study identified the upregulation of Nectin2 expression in NB patients and its correlation with advanced tumor staging. There is a consistent trend of increased Nectin2 expression being associated with poorer prognosis among publicly available datasets. Functional analysis showed that knockdown of *Nectin2* induces apoptosis of NB cells potentially through regulation of ANXA2 level.

Neuroblastoma is a pediatric cancer derived from primordial neural crest precursors and is the most common extracranial solid tumor seen in children. NB has great proliferative potential, resistance to apoptosis and high biological heterogeneity, accounting for variable clinical responsiveness. Thus, standard NB treatment consists of a combined multimodal approach, including chemotherapy, surgery, radiotherapy and immunotherapy [16]. NB patients have a poor prognosis and may develop resistance to conventional therapy [17]. Indeed, long-term survival rates for high-risk NB patients are currently <50%, despite multidisciplinary treatment [18]. As a result, there is a pressing need for the identification of novel treatments. Our previous research showed that tumor recurrence and progression are the main causes of death in children with NB [5]. In recent years, there have been many reports of various cell adhesion molecules expressed in NB tissues and cell lines that influence the development and prognosis of NB [ 19, 20] . Nectin2 belongs to the immunoglobulin superfamily and mediates many tumor-related processes, including cell proliferation, migration and invasion. According to the literature, Nectin2 is highly expressed in NB cells [21], but the relevant molecular mechanisms remain unknown. In the present study, 38 venous blood samples were collected from NB patients and 33 from healthy controls in our hospital. Nectin2 was shown to be highly expressed in the serum of NB patients and correlated with advanced tumor staging. Additional scrutiny of public datasets revealed that high Nectin2 expression is associated with poor NB prognosis. Although we detected elevated levels of Nectin2 in the serum of NB patients, enhancement of serum Nectin2 levels does not necessarily indicate Nectin2 overexpression in neuroblastoma. Molfetta *et al*. [22] demonstrated that Nectin2 is ubiquitinated on tumor cells and that this modification can promote protein degradation. However, no further studies have reported whether Nectin2 can undergo enzymatic hydrolysis or other degradation methods. The high level of serum Nectin2 may be caused by shedding of the Nectin2 ectodomain. Therefore, detailed information on which Nectin2 region the anti-Nectin2 antibody recognizes is crucial to explore the reason for the increased serum Nectin2 level. In addition, Nectin2 has two splice variants, Nectin2α and Nectin2δ, which have a common ectodomain and different cytoplasmic domains [23], and in our study, the antibody used in ELISA recognized human Nectin-2α. Which factor increases serum Nectin2 levels and which Nectin2 region the antibody recognizes have not been demonstrated in our study. In further studies, Nectin2 expression in the tumor tissue of NB patients will be determined to explore the reason for the increase in Nectin2. Meanwhile, whether Nectin2 undergoes ubiquitination degradation or lysosomal pathway degradation and enzymatic hydrolysis, resulting in the shedding of its fragments in serum, will be determined, and Nectin2 extracellular and intracellular peptides of different lengths will be designed to explore which region of Nectin2 is shed in serum.

Nectin2 expression was determined in NB cell lines by qRT-PCR and western blot analysis and was found to be highly expressed in SH-SY5Y and SK-N-BE2 cell lines. These results clearly indicate that *Nectin2* is an important gene in NB development, and further investigation of its function and mechanisms is warranted. High Nectin2 expression has also been reported in ovarian cancer, where it supports tumor cell adhesion, promoting tumor growth [24]. In addition, Oshima *et al*. [7] noted Nectin2 overexpression in breast and ovarian cancers and the antitumour activity of anti-Nectin2 mAbs. Judging from the above results, we hypothesized that Nectin2 may regulate the apoptosis, proliferation and migration of NB cells. Successful *Nectin2* knockdown was achieved by transfection of shRNA interference vectors into SH-SY5Y cells. Knockdown of *Nectin2* suppressed cell cycle progression, restraining cells in the G1 phase, inhibiting cell migration and promoting apoptosis. Low expression of E-cadherin and high expression of N-cadherin reduce cell adhesion ability and suppress endothelial-mesenchymal transition (EMT). Therefore, E-cadherin and N-cadherin have been considered marker proteins for the process of migration and invasion in many types of tumors. In the current study, we found that E-cadherin expression was increased and N-cadherin expression was decreased after *Nectin2* knockdown.

Investigations of downstream signaling following *Nectin2* knockdown included RNA-seq analysis, which revealed 258 genes with altered expression. Of particular interest, ANXA2 is known to be involved in NB [ 15, 25] and was downregulated upon *Nectin2* knockdown in SH-SY5Y cells. ANXA2 is a member of the annexin family of proteins that is expressed on the surface of various cancer cells and mediates intercellular and intracellular communication and cell survival [26]. ANXA2 has been found to be upregulated in many malignant tumors, including colorectal cancer, breast cancer, renal cell carcinoma, colorectal cancer, hepatocellular carcinoma and neuroblastoma [ 27– 29] . Indeed, ANXA2 may constitute an independent prognostic marker [30] and may interact with other proteins to influence tumor recurrence, progression and metastasis [31]. Wang *et al*. [15] reported that *ANXA2* knockdown induced apoptosis. In the current study, we found that *ANXA2* transfection into SH-SY5Y cells following *Nectin2* knockdown rescued the effect of *Nectin2* knockdown on apoptosis in SH-SY5Y cells.

Signal transducer and activator of transcription 3 (STAT3) is an oncogenic transcription factor that has been implicated in many human cancers and has emerged as a target for cancer therapy [32]. Odate *et al*. [33] showed that STAT3 inhibition decreased the tumor-initiating potential of NB cells and increased sensitivity to cisplatin. The rates of tumor growth declined, and the survival of tumor-bearing mice *in vivo* was prolonged. ANXA2 has previously been demonstrated to activate STAT3 in macrophages and enhance the formation of breast cancer, pancreatic cancer and hepatoma [34]. Indeed, ANXA2 overexpression promotes colorectal cancer invasiveness through the STAT3 pathway [35] and may regulate the proliferation, invasion and migration of colorectal cells through the same pathway [36]. STRING database analysis predicted that ANXA2 binds to STAT3, and thus, we speculate that STAT3 may be involved in the downstream signalling pathway of Nectin2 and ANXA2 during neuroblastoma pathogenesis. This hypothesis merits further exploration.

In conclusion, the current study provides insights into Nectin2 function, revealing its role in the apoptosis of NB cells, possibly via regulation of ANXA2 expression. Moreover, Nectin2 correlates with NB progression, indicating a worse prognosis for patients. We propose that Nectin2 may be a useful diagnostic biomarker and a potential therapeutic target for NB.

## Supporting information

091Supple

## Supplementary Data

Supplementary data is available at *Acta Biochimica et Biphysica Sinica* online.
