# Supplementary material for: Nectin2 influences cell apoptosis by regulating ANXA2 expression in neuroblastoma: Nectin2 influences SH-SY5Y cell migration and apoptosis
Source: Acta Biochim Biophys Sin (Shanghai). 2023 Mar 14;55(3):356–66. doi: 10.3724/abbs.2023020 (PMC10160223; doi:10.3724/abbs.2023020)
Supplement: 091Supple [file 091Supple.pdf]

Supplemental Data S1. Nectin2 shRNA lentivirus construction

Gene Name: Nectin2

Lentiviral Vector: GV493

Cloning site: *Age*I, *Eco*RI

Component order: hU6-MCS-CBh-gcGFP-IRES-puromycin

Vector Map

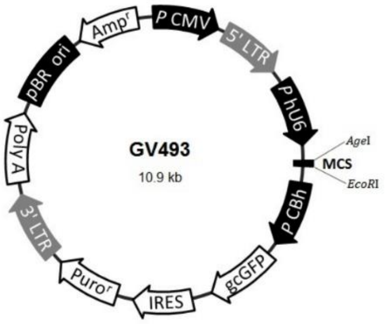

Sequencing results of positive clones and analysis of results:

CATCCAAACCTAAAAGATTACAAAAACAAATTACAAAAATTCAAAATTTTCGGGTTT
ATTACAGGGACAGCAGAGATCCAGTTTGGTTAATTAATCGAGCGGCCGCCCCCTTCA
CCGAGGGCCTATTTCCCATGATTCCTTCATATTTGCATATACGATACAAGGCTGTTAG
AGAGATAATTGGAATTAATTTGACTGTAAACACAAAGATATTAGTACAAAATACGTG
ACGTAGAAAGTAATAATTTCTTGGGTAGTTTGCAGTTTTAAAATTATGTTTTAAAATG
GACTATCATATGCTTACCGTAACTTGAAAGTATTTCGATTTCTTGGCTTTATATATCTT
GTGGAAAGGACGAAACACCGGGCAACTACACTTGCGAGTTTGCTCGAGCAAACTCGC
AAGTGTAGTTGCTTTTTGAATTCTCGACCTCGAGACAAATGGCAGTATTCATCCACGG
ATCCTAACCCGTGTCGGCTCCAACATAACTTACGGTAAATGGCCCGCCTGGCTGACCG
CCCAACGACCCCCGCCCATTGACGTCAATAGTAACGCCAATAGGGACTTTCCATTGAC
GTCAATGGGTGGAGTATTTACGGTAAACTGCCCACTTGGCAGTACATCAAGTGTATCA
TATGCCAAGTACGCCCCCTATTGACGTCAATGACGGTAAATGGCCCGCCTGGCATTGT
GCCCAGTACATGACCTTATGGGACTTTCCTACTTGGCAGTACATCTACGTATTAGTCA
TCGCTATTACCATGGTCGAGGTGAGCCCCACGTTCTGCTTCACTCTCCCCATCTCCCCC
CCCTCCCCACCCCCAATTTTGTATTTATTTATTTTTTAATTATTTTGTGCAGCGATGGG
GGCGGGGGGGGGGGGGGGGCGCGCGCCAGGCGGGGCGGGGCGGGGCGAGGGGCG
GGGCGGGGCGAGGCGGAAAGGTGCGGCGGCAGCCAATCAGAAGCGGCGCGCTCCAA
AGTTTCTTTTTATGCCAAGC

Supplemental Data S2. ANXA2 plasmid construction

Gene Name: ANXA2(NM_004039)

Cloning Vector: GV657

Component order: CMV enhancer-MCS-3flag-polyA-EF1A-zsGreen-sv40-puromycin

Cloning site: *Bam*HI / *Kpn*I

Vector Map

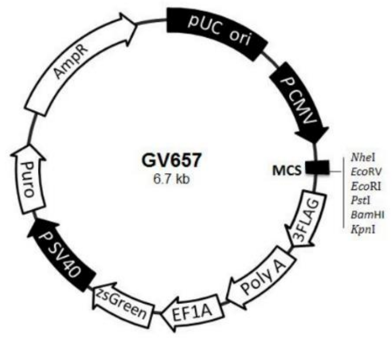

The plasmid that expresses ANXA2 was constructed by Genechem (Shanghai, China). Briefly, the linearized vector was obtained by digestion with restriction endonucleases. Acquisition of target gene fragments. Primers designed to construct the corresponding plasmids are

ANXA2(70643-1)-

p1:CACACTGGACTAGTGGATCCCGCCACCATGTCTACTGTTCACGAAATC;

ANXA2(70643-1)-

p2:AGTCACTTAAGCTTGGTACGTCATCTCCACCACACAGGTACAG.

The fragment of was amplified by PCR, using plasmid or bacterial liquid as the template source (98ºC 5 min; 98ºC 10 s, 55ºC 10 s, and 72ºC 90s, repeated for 30 cycles; 72°C 8 min). Then exchange of PCR products with vectors, the optimal molar ratio of linearized vector DNA and purified PCR product to add is 1:2. Recombinant products are directly transformed: Add 10 μL of exchange reaction product to 100 μL of competent cells, mix by flicking the tube wall a few times, and place on ice. Set aside for 30 minutes. Heat shock at 42°C for 90 s and incubate in ice-water bath for 2 min, add 500 μL LB medium and place at 37°CShaker for 1 h. Take an appropriate amount of bacterial liquid and spread it evenly on the plate containing the corresponding

antibiotics, in a constant temperature incubator. Inverted culture for 12-16 h. PCR identification (94ºC 3 min; 94ºC 30 s, 55ºC 30 s, and 72ºC 90s, repeated for 22 cycles; 72°C 5 min). The results of plasmid digestion showed that clone was a positive clone.

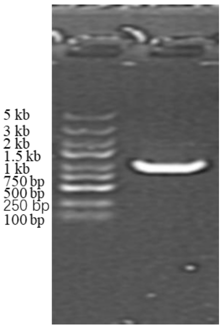

Sequencing results of positive clones and analysis of results:

CTGCACCTCGGTTCTTAATACGACTCACTATAGGGCTAGCGTTTAAACGGCCCTCTAG
ACTCGAGCGGCCGCCACTGTGCTGGATATCTGCAGAATTCCACCACACTGGACTAGTG
GATCCCGCCACCATGTCTACTGTTCACGAAATCCTGTGCAAGCTCAGCTTGGAGGGTG
ATCACTCTACACCCCCAAGTGCATATGGGTCTGTCAAAGCCTATACTAACTTTGATGCTG
AGCGGGATGCTTTGAACATTGAAACAGCCATCAAGACCAAAGGTGTGGATGAGGTCA
CCATTGTCAACATTTTGACCAACCGCAGCAATGCACAGAGACAGGATATTGCCTTCGCC
TACCAGAGAAGGACCAAAAAGGAACTTGCATCAGCACTGAAGTCAGCCTTATCTGGC
CACCTGGAGACGGTGATTTTGGGCCTATTGAAGACACCTGCTCAGTATGACGCTTCTG
AGCTAAAAGCTTCCATGAAGGGGCTGGGAACCGACGAGGACTCTCTCATTGAGATCAT
CTGCTCCAGAACCAACCAGGAGCTGCAGGAAATTAACAGAGTCTACAAGGAAATGTA
CAAGACTGATCTGGAGAAGGACATTATTTCGGACACATCTGGTGACTTCCGCAAGCTG
ATGGTTGCCCTGGCAAAGGGTAGAAGAGCAGAGGATGGCTCTGTCATTGATTATGAAC
TGATTGACCAAGATGCTCGGGATCTCTATGACGCTGGAGTGAAGAGGAAAGGAACTGA
TGTTCCCAAGTGGATCAGCATCATGACCGAGCGGAGCGTGCCCCACCTCCAGAAAGTA
TTTGATAGGTACAAGAGTTACAGCCCTTATGACATGTTGGAAAGCATCAGGAAAGAGG
TTAAAGGAGACCTGGAAAATGCTTTCCTGAACCTGGTTCAGTGCATTCAGAACAAGCC
CCTGTATTTTGCTGATCGGCTGTATGACTCCATGAAGGGCAAGGGGACGCGAGATAAG
GTCCTGATCAGAATCATGGTCTCCCGCAGTGAAGTGGACATGTTGAAAATTAGGTCTG
AATTCAAGAGAAAGTACGGCAAGTCCCTGTACTATTATATCCAGCAAGACACTAAGGG
CGACTACCAGAAAGCGCTGCTGTACCTGTGTGGTGGAGATGACGTACCAAGCTTAAGT
GACTACAAGGATGACGATGACAAGGATTACAAAGACGACGATGATAAGGACTATAAGG
ATGATGACGACAAATCTAGATAGTTAATTAAACCGGTAATAAAATATCTTTATTTTCATTA
CATCTGTGTGTTGGTTTTTTGTGTGAATCGATAGTACTAACATACGCTCTCCATCAAAAC
AAAACGAAACAAAACAAACTAGCAAAATAGGCTGTCCCCAGTGCAAGTGCAGGTGCC
AGAACATTTCTCTCTCGAGTCCATCGATACTAGTAAGGATCTGCGATCGCTCCGGTGCC
CGTCAGTGGGCAGAGCGCACAT.

Supplementary Table S1. The sequences of the primers used in this study

Gene	Primer sequence
Nectin2	F: 5'-GAGGACGAGGGCAACTACAC-3'<br>R: 5'-AGGGATGAGAGCCAGGAGAT-3'
ANXA2	F: 5′-CTCTACACCCCCAAGTGCAT-3′<br>R:5′-TCAGTGCTGATGCAAGTTCC-3′
ANXA2P2	F: 5′-GTTAAAGGAGACCTGGAAAATGCT-3′<br>R: 5′-GGTAGTCGCCCTTAGTGTCTTG-3′
GAPDH	F: 5'-TGCACCACCAACTGCTTAG-3'<br>R: 5'-GATGCAGGGATGATGTTC-3'
NEBL	F: 5'-GTGATATGGAGGTGGGAGAG-3'<br>R: 5‘ TTAGTTGCGAGGAAAGGGT-3'
ANXA2P2	F:5'-CATTGTGACCAACCGCGACA-3'<br>R:5'-GCCCAAAATCACCGTCTCCAG-3'
HRK	F: 5'-AGGTTGGTGAAAACCCTGTG-3'<br>R: 5'-TTTCTACGATCGCTCCAGGC-3'
RAB40B	F: 5′-TGGTCTTTTGACGGCATTGAT-3'<br>R: 5′-ATGTTGAAATTGCACAGAGGGC-3'
CDKN1A	F: 5′-TGTCCGTCAGAACCCATGC-3′<br>R: 5′-AAAGTCGAAGTTCCATCGCTC- 3′
VGF	F: 5' GGAACTGCGAGATTTCAGTCC-3'<br>R: 5' GTGCGGGTTTCCGTCTCTG-3'
GDF15	F: 5'- GCTCTCAGATGCTCCTGGTG-3'<br>R:5'-CGCAACTCTCGGAATCTGGA-3'
PER1	F:5′-CTGCTACAGGCACGTTCAAG-3′<br>R:5′-CTCAGGGACCAAGGCTAGTG-3′
UBALD1	F: 5′-TCATGATCAACCAGTTCGTGCTGAC-3′<br>R: 5′-TGGTGGTGATGGTGGCTGTAGG-3′

F: forward; R: reverse.

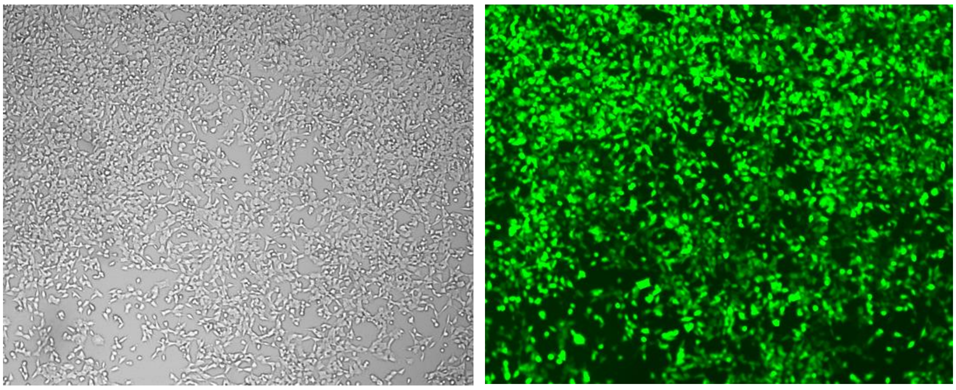

Supplementary Figure S1. Representative images of SH-SY5Y cells 48 h after ANXA2 overexpression plasmid transfection (>90% efficiency)
